# Cancer network activity associated with therapeutic response and synergism

**DOI:** 10.1186/s13073-016-0340-x

**Published:** 2016-08-24

**Authors:** Jordi Serra-Musach, Francesca Mateo, Eva Capdevila-Busquets, Gorka Ruiz de Garibay, Xiaohu Zhang, Raj Guha, Craig J. Thomas, Judit Grueso, Alberto Villanueva, Samira Jaeger, Holger Heyn, Miguel Vizoso, Hector Pérez, Alex Cordero, Eva Gonzalez-Suarez, Manel Esteller, Gema Moreno-Bueno, Andreas Tjärnberg, Conxi Lázaro, Violeta Serra, Joaquín Arribas, Mikael Benson, Mika Gustafsson, Marc Ferrer, Patrick Aloy, Miquel Àngel Pujana

**Affiliations:** 1 Breast Cancer and Systems Biology Lab, Program Against Cancer Therapeutic Resistance (ProCURE), Catalan Institute of Oncology (ICO), Bellvitge Institute for Biomedical Research (IDIBELL), Gran via 199, L’Hospitalet del Llobregat, Barcelona, 08908 Catalonia Spain; 2 Joint IRB-BSC-CRG Program in Computational Biology, Institute for Research in Biomedicine (IRB Barcelona), The Barcelona Institute of Science and Technology, Baldiri Reixac 10, Barcelona, 08028 Catalonia Spain; 3 Division of Preclinical Innovation, National Center for Advancing Translational Sciences (NCATS), National Institutes of Health, 9800 Medical Center Dr. Rockville, Bethesda, MD 20850 USA; 4 Experimental Therapeutics Group, Vall d’Hebron Institute of Oncology (VHIO), Cellex Center, Natzaret 115-117, Barcelona, 08035 Catalonia Spain; 5 Cancer Epigenetics and Biology Program (PEBC), IDIBELL, Gran via 199, L’Hospitalet del Llobregat, Barcelona, 08908 Catalonia Spain; 6 Department of Physiological Sciences II, School of Medicine, University of Barcelona, Feixa Llarga s/n, L’Hospitalet del Llobregat, Barcelona, 08908 Catalonia Spain; 7 Catalan Institution for Research and Advanced Studies (ICREA), Passeig Lluís Companys 23, Barcelona, 08010 Catalonia Spain; 8 Department of Biochemistry, Autonomous University of Madrid (UAM), Biomedical Research Institute “Alberto Sols” (Spanish National Research Council (CSIC)-UAM), Hospital La Paz Institute for Health Research (IdiPAZ), Arzobispo Morcillo 4, Madrid, 28029 Spain; 9 MD Anderson International Foundation, Arturo Soria 270, Madrid, 28033 Spain; 10 The Centre for Individualized Medicine, Department of Clinical and Experimental Medicine, Linköping University, Linköping, 58183 Sweden; 11 Hereditary Cancer Program, ICO, IDIBELL, Gran via 199, L’Hospitalet del Llobregat, Barcelona, 08908 Catalonia Spain; 12 Preclinical Research Program, VHIO, Cellex Center, Natzaret 115-117, Barcelona, 08035 Catalonia Spain; 13 Department of Biochemistry and Molecular Biology, Medical School Building M, Autonomous University of Barcelona, Bellaterra, 08193 Catalonia Spain

**Keywords:** Cancer, Network, Therapy, Synergy

## Abstract

**Background:**

Cancer patients often show no or only modest benefit from a given therapy. This major problem in oncology is generally attributed to the lack of specific predictive biomarkers, yet a global measure of cancer cell activity may support a comprehensive mechanistic understanding of therapy efficacy. We reasoned that network analysis of omic data could help to achieve this goal.

**Methods:**

A measure of “cancer network activity” (CNA) was implemented based on a previously defined network feature of communicability. The network nodes and edges corresponded to human proteins and experimentally identified interactions, respectively. The edges were weighted proportionally to the expression of the genes encoding for the corresponding proteins and relative to the number of direct interactors. The gene expression data corresponded to the basal conditions of 595 human cancer cell lines. Therapeutic responses corresponded to the impairment of cell viability measured by the half maximal inhibitory concentration (IC_50_) of 130 drugs approved or under clinical development. Gene ontology, signaling pathway, and transcription factor-binding annotations were taken from public repositories. Predicted synergies were assessed by determining the viability of four breast cancer cell lines and by applying two different analytical methods.

**Results:**

The effects of drug classes were associated with CNAs formed by different cell lines. CNAs also differentiate target families and effector pathways. Proteins that occupy a central position in the network largely contribute to CNA. Known key cancer-associated biological processes, signaling pathways, and master regulators also contribute to CNA. Moreover, the major cancer drivers frequently mediate CNA and therapeutic differences. Cell-based assays centered on these differences and using uncorrelated drug effects reveals novel synergistic combinations for the treatment of breast cancer dependent on PI3K-mTOR signaling.

**Conclusions:**

Cancer therapeutic responses can be predicted on the basis of a systems-level analysis of molecular interactions and gene expression. Fundamental cancer processes, pathways, and drivers contribute to this feature, which can also be exploited to predict precise synergistic drug combinations.

**Electronic supplementary material:**

The online version of this article (doi:10.1186/s13073-016-0340-x) contains supplementary material, which is available to authorized users.

## Background

Understanding of the molecular determinants of cancer therapeutic response has been greatly enhanced in recent years [1, 2]. For instance, defined transcriptional profiles have proved to be good predictors of treatment benefit [3]. In turn, specific alterations have been demonstrated to mediate the existence and/or promote the acquisition of resistance [4]. Globally, the integration of molecular data has been shown to be useful in predicting therapeutic sensitivity versus resistance [5–9]. However, despite these advances, cancer patients all too frequently show no or only modest benefit from a given therapy. The persistence of this fundamental clinical problem is partially attributed to the lack of specific biomarkers [10, 11]; nonetheless, the identification of a comprehensive measure of cancer cell activity could complementarily support the interpretation of therapy efficacy [12].

Cancer mutations portray cellular phenotypes reliant on the coordinated activity of specific biological processes and signaling pathways embedded in complex molecular networks [13–16]. During carcinogenesis, molecular networks show extensive rewiring and increased signaling entropy [17–19], which likely contribute to the characteristic robustness of the disease [20, 21]. Interestingly, increased signaling entropy has been associated positively with node connectivity and negatively with cancer survival and therapeutic benefit [22–24]. In parallel, cellular responses to combinations of molecular perturbations can be predicted based on how the corresponding targets are connected in the network [25, 26]. Thus, prediction of therapeutic response may be complemented by the inclusion of a potentially comprehensive cellular measure that integrates diverse levels of molecular data. In addition, searching for maximal systems-level perturbations induced by drugs may help to identify synergistic therapeutic combinations. Such analyses could help to identify combinations that are more effective than single-compound approaches. This study assesses these hypotheses using a weighted network measure based on the feature of network communicability [27, 28].

## Methods

### Interactome network analyses

Release #9 of the Human Protein Reference Database (HPRD) [29], which contains 9670 proteins and 39,172 interactions (9267 and 36,893 in the main interactome network component, respectively), and a high-quality human protein interaction network derived from the combination of reliable literature-curated binary interactions and well-verified high-throughput yeast two-hybrid interactions (7401 proteins and 20,614 interactions; 6993 and 19,009 in the main component, respectively) [30] were analyzed in this study. The corresponding main components were used for subsequent analyses, excluding proteins with no assigned Entrez identifier and homodimers. The significance of the results was also assessed using a random undirected network that was constructed applying the latmio_und function (Brain Connectivity Toolbox in Python), which preserves the node degree distribution and network connectedness.

### Gene expression, therapeutic responses, and cancer cell line data

Normalized expression data from 595 cancer cell lines, for which the associated drug sensitivity data were available, were obtained from the corresponding repository [8]. If > 1 microarray probes represented a given gene, the probe with the highest standard deviation was used in subsequent analyses. For gene products not represented in the microarray (n = 1428 in HPRD), the average expression value for all measured genes was assigned. The normalized IC_50_ for 130 unique drugs, together with information on the mutational status of given proto-oncogenes and tumor suppressor genes, were obtained from the original study [8]. Additionally, each drug was categorized as “single node” or “multiple node” perturbations, on the basis of current knowledge of the respective mechanisms of action (Additional file 1). Genetic, genomic, and molecular alterations identified in cancer cell lines were taken from the same repository [8]; the alterations examined for global correlation with CNA were of *ERBB2* or *MYC* amplification, and *BRAF*, *EGFR*, *PIK3CA*, *PTEN*, *RB1*, or *TP53* mutation. CNA was also evaluated for its global correlation with protein expression using normalized reverse phase protein array (RPPA) data for 81 cell lines obtained from The Cancer Proteome Atlas [31].

### Cancer network activity algorithm

CNA was defined following the concept of “weighted communicability” [28]. First, for each network edge (i.e. protein-protein interaction in the network) a weighted expression-based value was obtained as follows: ${w}_{ij}=\frac{x_j}{{\displaystyle {\sum}_{k\in {U}_i}{x}_k}}\cdotp {x}_i+\frac{x_i}{{\displaystyle {\sum}_{k\in Uj}{x}_k}}\cdotp {x}_j$, where *w*_*ij*_ is the weighted value of the edge that connects nodes (i.e. gene products or proteins) *i* and *j*, *x*_*i*_, *x*_*j*_ are the expression values of the corresponding genes, and *U*_*i*_ and *U*_*j*_ are the sets of their corresponding direct interactors (*k*). Therefore, the ratio $\frac{x_j}{{\displaystyle {\sum}_{k\in {U}_i}{x}_k}}$ computes the relative expression of interactor *j* in the direct neighborhood of *i* (*U*_*i*_) and the product $\frac{x_j}{{\displaystyle {\sum}_{k\in {U}_i}{x}_k}}\cdotp {x}_i$ computes the expression of *i* proportional to the expression of *j* in *U*_*i*_. The distribution of the expression of *i* according to its interactors (*U*_*i*_) satisfies that ${\displaystyle {\sum}_{j\in {U}_i}\frac{x_j}{{\displaystyle {\sum}_{k\in {U}_i}{x}_k}}=\frac{{\displaystyle {\sum}_{j\in {U}_i}{x}_j}}{{\displaystyle {\sum}_{k\in {U}_i}{x}_k}}}=1$ and therefore ${\displaystyle {\sum}_{j\in {U}_i}\frac{x_j}{{\displaystyle {\sum}_{k\in {U}_i}{x}_k}}\cdotp {x}_i={x}_i}$. The preprocessed and normalized gene expression values were obtained from the original cancer cell line study [8]. Subsequently, a matrix (*W*) of weighted edges was generated by compiling all *w*_*ij*_ values, where (*i,j*) belong to the complete set of edges (*E*) in the main network component. In this analysis, *w*_*ij*_ values were normalized by row and column weights using the product of $\sqrt{d_i{d}_j}$ , where *d*_*i*_ = ∑_*j*_*w*_*ij*_, which therefore corrects for the potential bias introduced by nodes with many interactors and large weights [28, 32]. In *W*, the centrality measure of a given node *i* was defined by computing all paths that start and finish at *i*; *C*(*i*) = (*e*^*W*^)*ii*, where ${e}^W={\displaystyle \sum_{k\ge 0}\frac{W^k}{k!}}$. Thus, *W*^*k*^ represents the *k*^*th*^-power of the weighted adjacency matrix given all paths of length *k* and longer paths are penalized by including the factorial *1/k!*. Finally, a global measure of network (G) activity (CNA) can be defined by the sum of node centralities, as follows: $CNA(G)={\displaystyle \sum_{i\in V}C(i)}$. The normalized *x* ∈ (0, 1) CNA score (HPRD-based, Additional file 2) was obtained by computing $\frac{x-a+1}{b-a+1}$, where *a* and *b* are the minimum and maximum of observed CNA values, respectively. The significance of the CNA-drug/therapeutic feature associations was computed empirically by performing 1000 permutations of the CNA-cell line identities.

### Gene ontology and pathway annotation analyses

The Gene Ontology (GO) Biological Processes term annotations were downloaded from the Open Biological Ontologies release 2012/06 (MySQL version). Genes annotated at level 5 or lower in the hierarchy were assigned to level 4, but those also occurring at level 3 were excluded. Only those terms with a frequency of ≥ 5 % in the analyzed protein sets were evaluated. REACTOME pathway annotations were downloaded from the corresponding repository (www.reactome.org). Statistical significance of term/pathways was assessed using 2 × 2 contingency tables and Fisher’s exact tests. Values of *p* < 0.05 after false-discovery rate (FDR) [33] correction were considered significant.

### Transcription factor analyses

Professional TRANSFAC predictions (version December 2014) for 599 TF were used for the enrichment analysis. In the HRPD dataset, 4661 genes were regulated by any TF and 836 nodes corresponded to the “high centrality” set. Each TF was tested for its enrichment in this set using Fisher’s exact test. If > 1 microarray probe was present for a given TF in the cancer cell lines dataset [8], the average expression value was used for the correlation analysis with the drug IC_50_ values.

### Cell culture

All cell lines used in this study were cultured following standard protocols and cellular viability was evaluated by performing assays based on methylthiazol tetrazolium (MTT, Sigma-Aldrich). The human mammary epithelial cell line MCF10A (ATCC) was used as the non-tumorigenic control. The drugs were purchased from Sigma-Aldrich (metformin) and from LC Laboratories. The reported results to at least four independent assays for each drug/combination and to quadruplicates for each data point.

### Synergism analysis

The non-additive effects of drug combinations were assessed using two methods: the Drug Combination Index (DCI) of the Chou-Talalay method [34] and a two-parameter algorithm based on Newton-Raphson’s methodology [35]. The DCI was defined as follows: $CI=\frac{D_1}{{\left({D}_{50}\right)}_1}+\frac{D_2}{{\left({D}_{50}\right)}_2}$, where *D*_1_ and *D*_2_ are the effects of drugs *1* and *2* in the combination causing 50 % viability, respectively, and (*D*_*50*_)_1_ and (*D*_*50*_)_2_ are the effects in the corresponding single-drug assays. Thus, the DCI was computed for a range of viability effects and values of ≤ 0.85 and ≥ 1.20 correspond to synergism and antagonism, respectively. The two-parameter algorithm was implemented as follows: given *n* drug doses *x* = (*x*_0_,…, *x*_n-1_) and *n* cellular responses *y* = (*y*_0_,…, *y*_n-1_), the method was applied to compute responses *ŷ* = (*ŷ*_0_, …, *ŷ*_*n* − 1_), *ŷ*_*i*_ = *f*_*u*_(*x*_*i*_), which minimize ‖*y* − *ŷ*‖^2^, where *f*_*u*_ is the unaffected ratio of cells, ${f}_u(x)=\frac{1}{1+{\left(\frac{x}{D_{50}}\right)}^m}$, and *D*_*50*_ and *m* (slope) are free parameters.

## Results

### An integrative analytical strategy

**Fig. 1:**
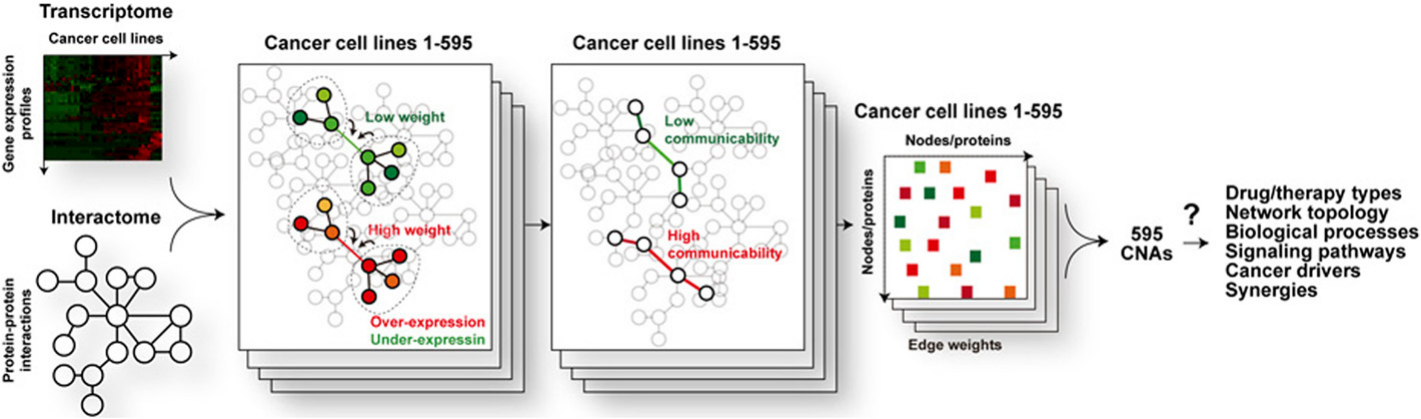
Strategy analysis. The basal gene expression of hundreds of cancer cell lines is integrated into the interactome network and a CNA score is then assigned to each cell line by computing a weighted adjacency matrix. Next, CNA measures are evaluated for their correlations with types of drugs or therapies, network topology, biological processes and signaling pathways, cancer drivers, and drug synergies

Genes and proteins are functionally organized within complex networks [14]. In cancer, biological processes and signaling pathways in such networks are often robust to perturbations [7, 17–19, 36, 37]. We hypothesized that a measure that integrates molecular interactions and expression levels could, at least partially, predict cancer therapeutic responses. We tested the hypothesis by first integrating the known human interactome network (i.e. network of protein-protein-interactions) with basal gene expression measurements in 595 cancer cell lines whose sensitivity (i.e. IC_50_ values) to 130 cancer drugs was determined [8]. In this approach (Fig. 1), starting with an undirected interactome network and for each node (protein) and edge (interaction), a weight is assigned to an edge as proportional to the expression level of the corresponding interaction partner and relative to the expression levels of the direct interactors (see “Methods”). Subsequently, the weighted adjacency matrix is used to apply the concept of network “communicability” [27, 28] as a prediction of cancer cell activity that may, in turn, be associated with specific cancer features and differences in therapeutic responses.

While whole-genome expression measurements in a single sample assume uncertainties in the values of some genes, the integrated gene expression-IC_50_ profiling dataset showed strong correlation with the tissue of origin, as well as with established cancer drivers [8]. To further evaluate the properties of this dataset at the systems level, we computed the expression correlations between any two genes that encode interacting proteins; thus, consistent with previous observations in health and disease [38], and compared to randomly chosen gene pairs, the genes that encode interacting proteins tend to be positively co-expressed (Pearson’s correlation coefficient (PCC) = 0.12; Wilcoxon rank test *p* = 6.0 × 10^−300^; Additional file 3). This validation supported the established framework for assessing the relevance of the newly defined measure of CNA.

### Associations between CNA and type of therapies

**Fig. 2:**
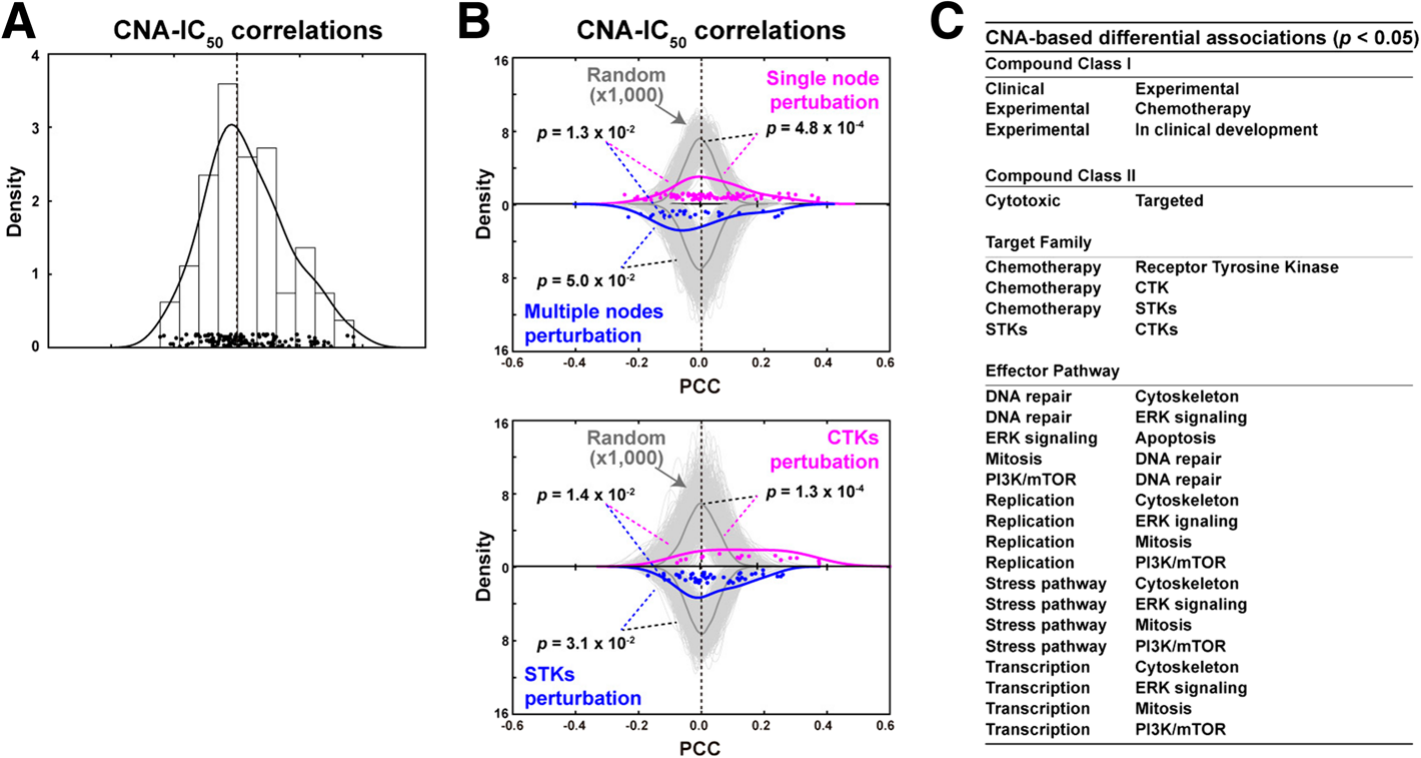
CNA-IC_50_ correlation discriminates drugs and therapies. **a** Global distribution of PCCs between CNA values and IC_50_ profiles for all cancer cell lines and drugs, respectively. **b** *Top panel*, distribution of PCCs for drugs that target a single network node (i.e. targeted therapies) versus drugs that target multiple nodes and/or broad processes. The Wilcoxon test *p* values for the comparison of distributions are shown; *gray distributions* correspond to random permutations of CNA and cancer cell line correspondences. *Bottom panel*, distribution of PCCs for drugs that target CTKs or STKs. **c** Significant differences (PCCs, empirical *p* < 0.05) between drugs originally assigned to a different Class, Targeted Family, or Effector Pathway [8]

First, we determined the correlations between the CNA values and IC_50_ profiles of different classes of drugs. While the overall distribution of PCCs was centered on zero (Fig. 2a), significant differences (*p* < 0.05 based on 1000 permutations of cell line identities) were found between distinct drug types or therapeutic strategies. The IC_50_ of drugs that target broad processes, such as those used in chemotherapy, tended to correlate negatively with CNA; that is, higher activity is associated with higher therapeutic sensitivity (i.e. lower IC_50_; Fig. 2b, top panel). Drug classification between single- and multiple-node perturbations is detailed in Additional file 1 and all other classifications were obtained from the original study [8]. This result appears to be consistent with the known association between cell proliferation and response to chemotherapy [39].

In contrast to chemotherapies, the IC_50_ of drugs that define targeted therapies tended to correlate positively with CNA, and significant differences were observed among drugs of this type; for example, between drugs that target cytoplasmic (non-receptor) tyrosine kinases (CTKs) and S/T kinases (STKs) (Fig. 2b, bottom panel). Importantly, the results were replicated with a high-quality human protein interaction network (Additional file 4), but the association disappeared using a random network model that preserved the original degree distribution and connectedness (Additional file 5). Overall, significant differences (empirical *p* < 0.05) were found in terms of previously defined Compound Class, Target Family, and Effector Pathway (Fig. 2c) and, remarkably, none of these associations was detected using the null model (*p* values > 0.05). Together, these observations indicate that CNA captures biologically relevant information linked to cancer therapeutic responses.

### Biological processes and pathways influencing CAN-therapeutic associations

**Fig. 3:**
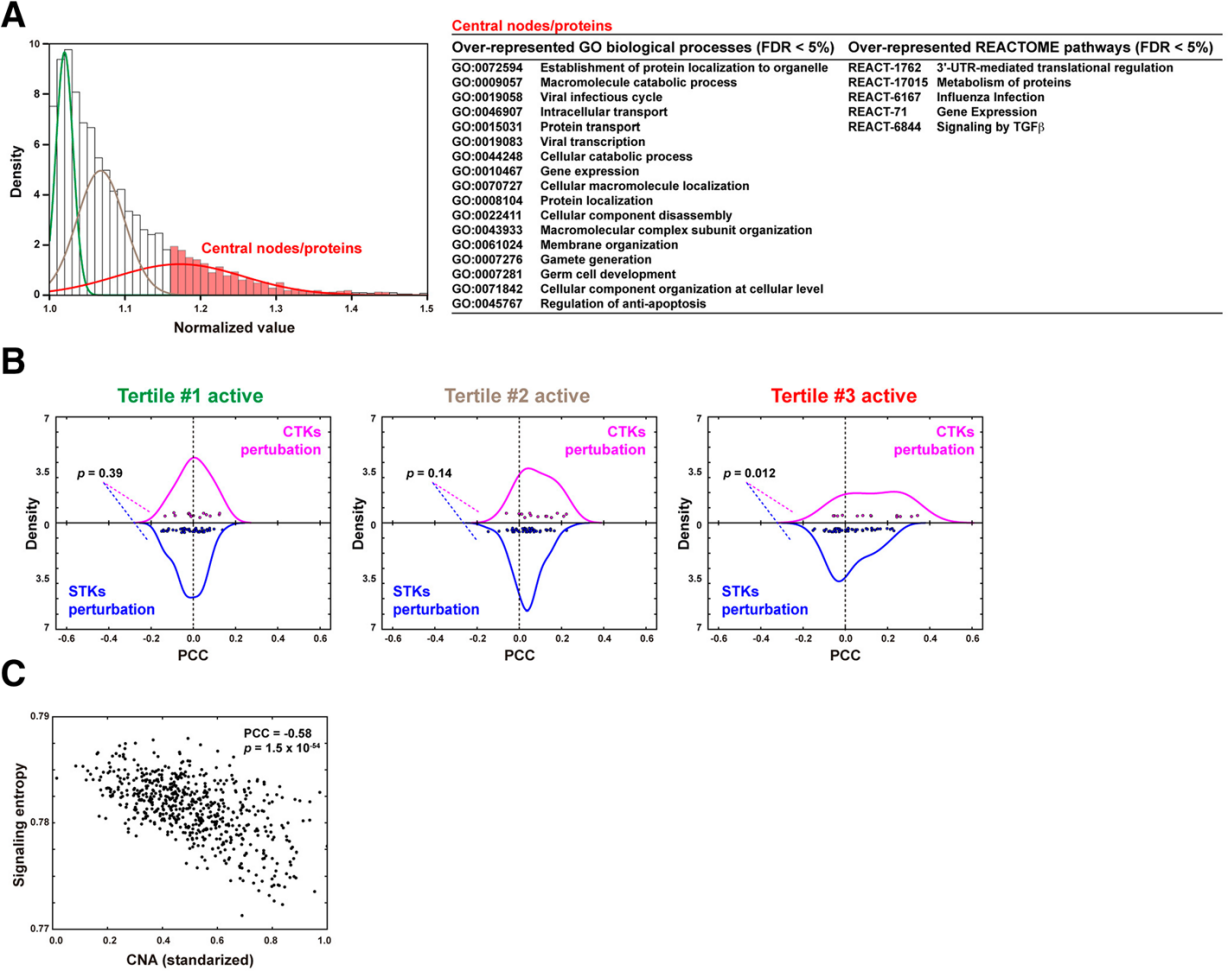
Specific biological processes and high-centrality network nodes contribute to CNA-associated differences. **a** Tertile distributions of network nodes according to standardized centrality measure. The GO biological processes and REACTOME pathways significantly enriched (FDR < 5 %) in the tertile with the highest centrality are listed in the right panels. **b** *Panels* showing the distribution of PCCs for drugs that target CTKs or STKs, when the lowest, middle, or highest tertiles of centrality are active (i.e. nodes for the remaining tertiles are “deactivated” by assigning the average network centrality value). The empirical *p* values for the comparison of distributions are shown; distributions are only different when the highest tertile of centrality is active, as seen for the complete dataset. **c** Correlation between signaling entropy and CNA measures in the same cancer cell lines dataset

To determine which biological processes and signaling pathways were contributing to the associations observed above, we examined the distribution of node centrality in CNAs from all cell lines. This was done using the measure of all possible weighted network paths starting and ending at the corresponding node (i.e. protein). The highest tertile of centrality included nodes that showed an over-representation of Gene Ontology (GO) terms related to a range of biological processes, including apoptosis, gene expression, and immune response (Fig. 3a). Furthermore, this set of edge-weighted central nodes showed significant over-representation of members of the TGFβ signaling pathway (Fig. 3a).

Interestingly, the set of central nodes was found to be responsible for the differential correlations between CNA and IC_50_ profiles. For example, in the case of drugs targeting CTKs or STKs, skewed distributions were evident only when the high-centrality node set remained “active” (node “inactivation” was achieved by assigning an average value of centrality from the complete main network component; Fig. 3b, right panel). The mid-centrality node set showed a trend for differences, but statistical significance was not reached (Fig. 3b, middle panel). Therefore, key cancer-associated biological processes and pathways that occupy central positions in the network contribute to the association between CNA and therapeutic responses.

The above observations are similar to independent results obtained using an analogous network measure, signaling entropy [37, 40]. This measure is derived from a sample-specific integration of gene expression profiles in protein interactome data and provides an estimation of signaling promiscuity [37, 40]. Interestingly, high entropy has been associated with over-expression of hub proteins, over-expression of common pathways to above (e.g. immune response), and poor prognosis and therapeutic resistance [17, 18, 22–24]. Given these observations, we assessed the correlation between CNA and entropy [24] and detected a strong negative correlation (PCC = –0.58, *p* = 1.5 × 10^−54^; Fig. 3c).

Consistent with the negative correlation with entropy, and using the complete cell line collection, CNA was found to be negatively correlated with *MYC* amplification (Spearman’s correlation coefficient (SCC) = –0.13, FDR-adjusted *p* = 0.049) and with annexin 1 (ANXA1) and caveolin 1 (CAV1) protein expression detected in RPPA assays (SCCs = –0.50 and –0.67, FDR-adjusted *p* = 0.003 and 0.0001, respectively). Notably, over-expression of ANXA1 and CAV1 has been associated with therapeutic resistance in different cancer settings [41, 42]. Overall, these observations are coherent with CNA-drug associations and further suggest that CNA captures biologically meaningful information for the comprehensive understanding of therapeutic responses.

### Cancer drivers influence CNA*-*therapeutic associations

**Fig. 4:**
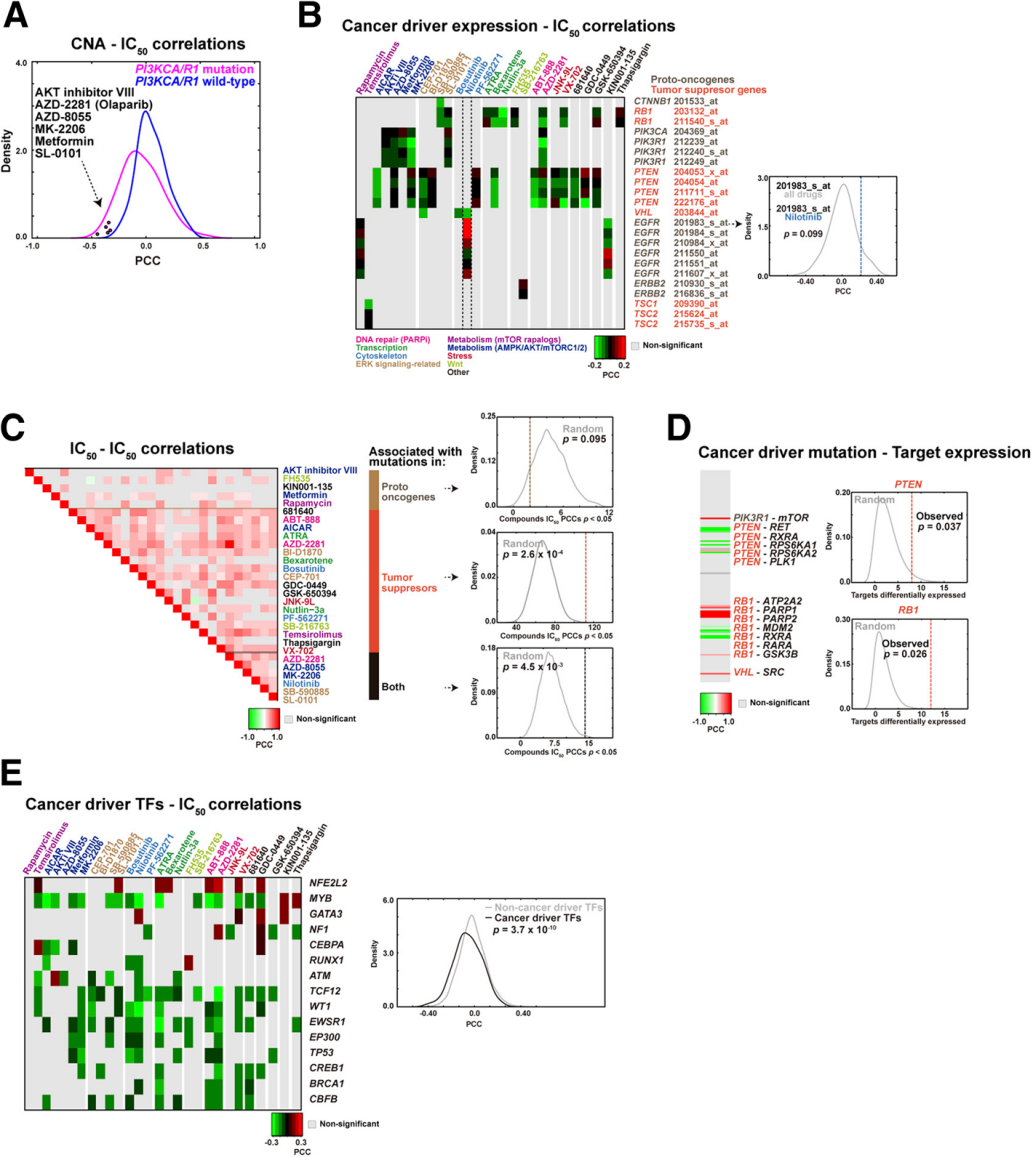
Assessment of CNA-drug IC_50_ associations. **a** Distributions of PCCs for cancer cell lines with mutated or wild-type *PIK3CA/R1*. The drugs contributing to the negative correlation bias for *PIK3CA/R1*-mutated cell lines are listed. **b** *Left panel*, *heatmap* showing the results of the PCC analysis between the IC_50_ profiles of the identified drugs (based on differential CNA*-*IC_50_ correlations and mutational status) and the expression profiles of proto-oncogenes and tumor suppressors (by microarray probe; the results for nilotinib are marked). The significant (empirical *p* < 0.05) correlations are shown. *Drugs* are color-coded according to the corresponding molecular or biological process target. *Right panel*, results for the *EGFR* 201983_s_at probe correlation with all drug IC_50_ values (distribution) or with nilotinib IC_50_ (*brown lane*), originally identified as associated with CNA. **c** *Left panel*, *heatmap* showing the unsupervised clustering of PCCs between the IC_50_ profiles of associated drugs (based on CNA) with the mutational status of proto-oncogenes or tumor suppressors, or both. *Right panels*, comparison of the observed number of correlated effects (*vertical lines*) against equivalent random sets of drugs (distributions). The empirical *p* values are shown. **d** *Left panel*, *graph* showing the targets of CNA-IC_50_-based drugs that are differentially expressed between mutated and wild-type cancer cell lines for each of the proto-oncogenes or tumor suppressors analyzed. *Red* and *green* indicate over-expression and under-expression in the corresponding mutated setting, respectively. *Right panels*, comparison of the observed number of differentially expressed targets in *PTEN* or *RB1* mutated cell lines (*vertical lines*) against equivalent random sets of cancer cell lines. The empirical *p* values are shown. **e** *Left panel*, *heatmap* showing the results of the PCC analysis between the expression of cancer driver TFs and the IC_50_ profiles of drugs associated (based on CNA) with the mutational status of proto-oncogenes and/or tumor suppressors. *Right panel*, PCC distributions for cancer driver TFs (excluding *CTNNB1* and *PTEN*) and the rest of human TFs according to TRANSFAC annotations. The *p* value of the Wilcoxon rank test is shown

Since CNA discriminates types of cancer drugs and key cancer processes/pathways mediate the observed differences, we next assessed associations according to the mutational status of known proto-oncogenes and tumor suppressors. The comparison of wild-type and mutated settings revealed differential distributions of CNA-IC_50_ correlations for the *CTNNB1*, *EGFR*, *ERBB2*, and *PIK3CA* (analyzed jointly with *PIK3R1*) proto-oncogenes and for the *PTEN*, *RB1*, *TSC1/2*, and *VHL* tumor suppressors (Fig. 4a shows results for *PIK3CA/PIK3R1* status; and Additional files 6 and 7).

Several observations support the systems-level relevance of the observed differences by cancer driver gene type and its mutational status. First, most (26/28, 93 %) of the CNA-IC_50_ differential associations were concordant with the corresponding gene expression-IC_50_ correlations (PCC *p* values < 0.05; Fig. 4b, left panel); therefore, CNA frequently captures the global network impact of a precise cancer driver. Interestingly, among these drugs, nilotinib IC_50_ was found to represent the strongest positive correlation with *EGFR* expression, but the CNA-nilotinib IC_50_ correlation in *EGFR* mutants was negative (Additional file 6) and there is preclinical evidence of synergism between this drug and anti-EGFR targeted therapy [43]. In addition, CNA was found to be correlated with the expression (probe level) of 21/28 (75 %) of the specific drug targets (Additional file 8). Further supporting the proposed influence of key processes/pathways, the drugs identified using the CNA measure (i.e. drugs linked to differential distributions of CNA-IC_50_ correlations by driver mutational status, Additional file 6) were not the highest ranked when gene expression-IC_50_ correlations were analyzed (Fig. 4b, right panel). Second, there were indications of signaling pathway coherence. While the mutation profiles of *PIK3CA/R1* and *PTEN* were not correlated across cancer cell lines (as expected, PCC = −0.05), two significant CNA-IC_50_ concordances were found between the gene mutation settings: for AZD-2281 (olaparib), which targets PARP1/2 and the DNA damage response; and for AZD-8055, which targets mTORC1/2 (Additional file 6). Third, there were also indications of biological process coherence; the drugs targeting (based on CNA) the mutational status of proto-oncogenes were frequently found to alter metabolism (as Effector Pathway [8], Fisher’s exact test *p* = 0.014), whereas the drugs targeting the mutational status of tumor suppressor genes were frequently found to alter transcription (*p* = 0.001). Fourth, the drugs that target the mutation status of tumor suppressors were more frequently correlated with one another (for their IC_50_) than randomly expected (empirical *p* = 2.6 × 10^−4^), whereas an opposite trend (*p* = 0.095) was observed for drugs that target proto-oncogene mutations (Fig. 4c). There were target or effector pathway redundancies in the previous drug sets that could have produced bias in the analysis; however, while the number of drugs analyzed is relatively small, the effect is clearly different for tumor suppressors (Fig. 4c). Finally, the analyses of *PTEN* and *RB1* captured most of the differential CNA-IC_50_ associations, and the corresponding drug targets (i.e. the targets of the analyzed drug IC_50_) were frequently found to be differentially expressed in cancer cell lines harboring *PTEN* or *RB1* mutations (empirical *p* values < 0.05; Fig. 4d).

The above observations support that CNA captures biologically relevant information across different cancer settings. To characterize this link at a complementary network level, we examined whether known transcription factors (TFs) that act as cancer drivers [44] commonly regulate the set of nodes that determines CNA. Overall, there was a significant enrichment for predicted binding sites of the cancer driver TFs in the promoters of genes encoding the high-centrality set (odds ratio = 1.89, *p* = 0.007). Specifically, three of the 17 cancer driver TFs were found to be over-represented (as predicted binding sites: BRCA1, CBFP and EP300; Fisher’s test *p* values < 0.05). Next, an analysis of correlations between the expression of all cancer driver TFs and the drugs identified above (as linked to the mutational status of proto-oncogenes or tumor suppressors) revealed significant differences relative to non-cancer driver TFs (Wilcoxon rank test *p* = 3.7 × 10^−10^, excluding the coincident *CTNNB1* and *PTEN*; Fig. 4e). Collectively, these data further illustrate key system-level features captured by the CNA measure and linked to cancer therapeutic response.

### Using CNA evidence to predict synergism

Given the CNA associations involving specific drugs and therapeutic approaches, and key processes/pathways and cancer drivers, we hypothesized that this information could identify the combination of drugs that maximizes the perturbation of the cancer network. In turn, since interplay of cancer hallmarks is involved, maximizing the perturbation will potentially increase the likelihood of a synergistic effect. With this aim, we predicted that those pairs of drugs whose individual IC_50_ profiles were not correlated (in the specific mutated context) could provide greater network perturbation, thus achieving synergistic effects. An analogous rationale has recently been tested using multidimensional data modeling [45].

**Fig. 5:**
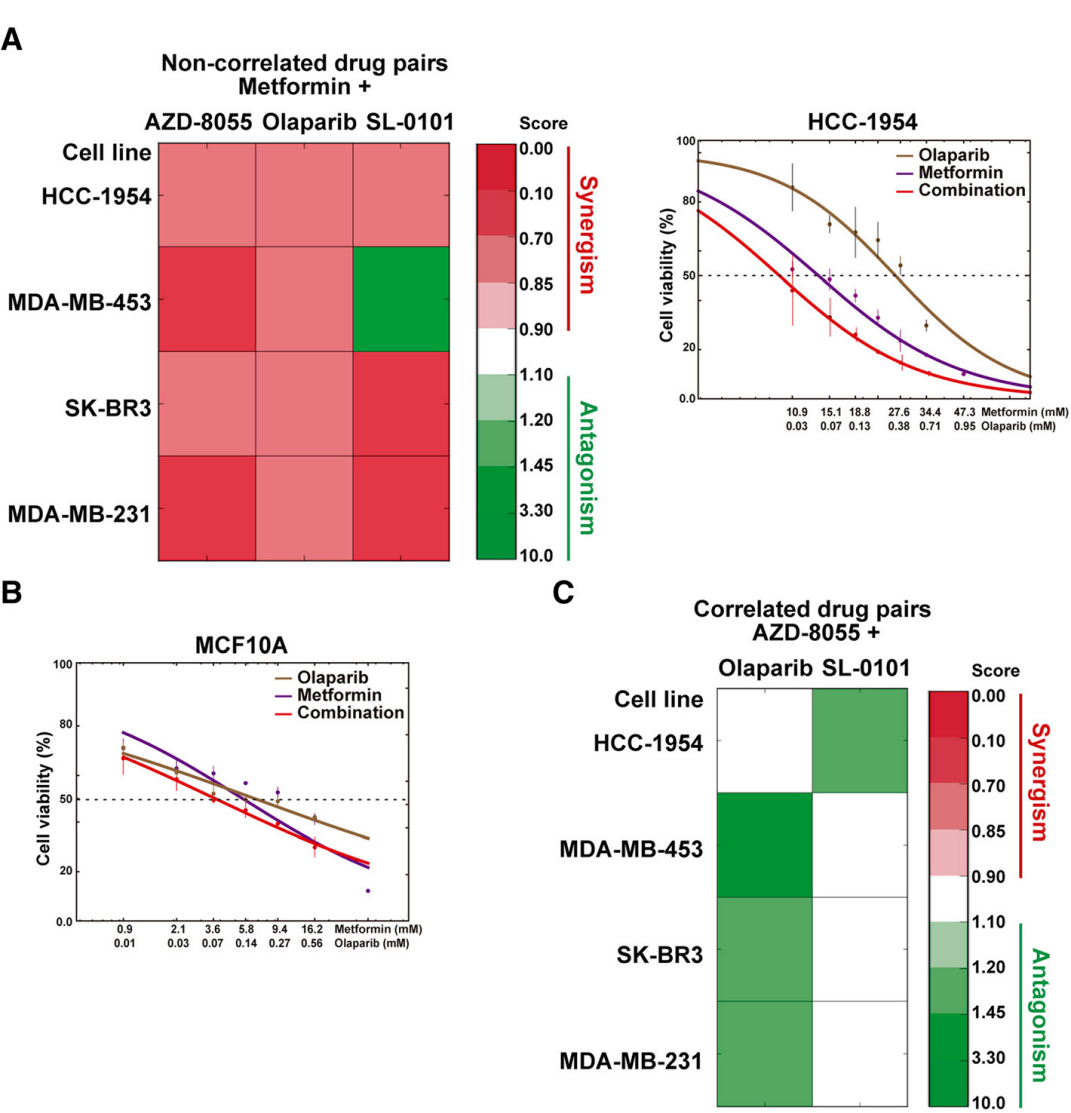
Assessment of the effect of drug pairs based on CNA evidence. **a** *Left panel*, *heatmap* showing the DCIs obtained by assessing metformin combinations with non-correlated (in the original dataset [8]) drugs in four breast cancer cell lines. *Right panel*, *graph* showing the results for metformin and/or olaparib in HCC-1954 cells. **b** *Graph* showing the results for metformin and/or olaparib in MCF10A cells. **c** *Heatmap* showing the DCIs obtained by assessing AZD-8055 combinations with correlated drugs

Following on from the above premises, the study was centered on PI3K-mTOR signaling, as this pathway plays a key role in carcinogenesis and therapy response [46, 47]. Of the drugs targeting the *PIK3CA/R1* status (Additional file 6), we selected metformin because its IC_50_ was not correlated (in *PIK3CA/R1* mutated cell lines) with AZD-8055, olaparib, or SL-0101 (ribosomal S6 kinase (RSK) inhibitor). The following cell lines were used to evaluate these combinations: MDA-MB-453 and HCC-1954, both of which harbor an oncogenic *PIK3CA* mutation; and MDA-MB-231 and SK-BR3, which harbor molecular alterations linked to increased AKT activity and PI3K-inhibition sensitivity [48]. Thus, the assessment of the inhibitory effect of each metformin combination revealed significant synergism in nine of 12 instances when compared to single-drug assays (DCI < 0.85; Fig. 5a). Consistent results were obtained in three independent assays of each combination and using an alterative analytical method based on two parameters (see “Methods”). In addition, assays with the non-tumorigenic breast cancer MCF10A cell line did not show synergisms (Fig. 5b shows results for the combination of two approved drugs: metformin and olaparib). Moreover, using the same cell lines, the assessment of drugs with positively correlated IC_50_ profiles did not reveal any synergism, but four of eight combinations/cell lines showed an antagonistic effect (Fig. 5c). Thus, the proportions of the observed effects between uncorrelated and correlated drug pairs were significantly different (Z-score *p* = 0.001). Finally, the identified synergistic combinations were tested in two additional breast cancer cell lines harboring oncogenic *PIK3CA* mutations: BT-474 and MCF7. The results showed similar trends as above except for two combinations in MCF7 (Additional file 9). Of note, BT-474 and HCC-1954 correspond to HER2-positive cancer subtype, and the efficacy of combining of metformin and olaparib would be consistent with PARP-inhibition leading to an increase of glycolysis by rising NAD+ and metformin counteracting this activity [49, 50]. In addition, the synergistic results are consistent with and expand on recent observations of the effect of combining PI3K/mTOR and PARP inhibitors in the treatment of *BRCA1*-related breast cancer [51, 52].

## Discussion

The understanding of cancer cell response to current standard therapies is fundamental for developing effective novel and/or combined approaches and, therefore, for improving the clinical management of cancer patients [1]. Since carcinogenesis is reliant on the interplay of several biological processes and signaling pathways [53], the understanding of therapeutic response may be improved by taking into account all of the potential molecular measures in a given cancer model [12]. In this study, we addressed this question by computing a weighted network score for hundreds of cancer cell lines and assessing its association with the cancer cell sensitivity profiles to more than one hundred experimental or approved drugs [8]. The results show that the newly defined measure of “cancer network activity” correlates with differences in the mode of action of the drug types. In turn, these differences are likely to be mediated by topologically central proteins and key cancer-associated biological processes and pathways. Complementarily, these observations may lead to additional studies focused on local topological network features that could detect cancer cell activity to some extent. However, the present study presents certain limitations that should be acknowledged. In particular, more accurate results may be obtained by including information on the correspondence between gene and protein expression levels, and by taking into account protein isoforms and key functional post-transcriptional and post-translational modifications. Interestingly, CNA is found to be negatively correlated with a global measure of signaling entropy [22–24, 40], which collectively may depict complementary systems-level views of cancer status. This observation is further extended by the negative correlation between CNA and biomarkers therapeutic resistance in different cancer settings (i.e. ANXA1 and CAV1) [41, 42].

The study goes beyond the novel observation of the relevance of CNA, suggesting that this property may be useful in predicting synergistic drug combinations. Given the existence of > 30,000 possible pair combinations among those drugs currently approved for cancer therapy, the value of data integration has previously been proposed [54]. Importantly, network topology can predict subtype-specific therapeutic targets [55] and, in turn, the cellular response to a given combination of compounds can predict the connectivity of the targets [26]. Critically, however, synergistic effects appear to be more dependent on the molecular context than single drug activities [56]. This observation is reminiscent of the complexity of genetic interactions [57] and might be expected if it is considered that the overall effect of combinations is mediated by the interplay of different processes and/or pathways. In our study, guided by differences in the correlation between CNA and IC_50_profiles, and based on the premise that drug IC_50_ profiles should not be correlated in order to likely reveal synergisms, we suggest that CNA is useful for identifying effective drug combinations. A recent comprehensive study has used a similar rationale to identify synergistic effects in a specific cancer context [45]. While our study was primarily aimed at evaluating the systems-level influence of cancer therapies, several algorithms specifically intended to predict synergism have been developed and evaluated [58, 59]. Some of these algorithms use pathway information and/or dissimilarity gene expression profiles for inference, but the integration of this information is not used for network modeling and the premise of dissimilar IC_50_ profiles for potential drug combinations is not considered. Larger analyses of drug pairs may be warranted to corroborate the established premises.

## Conclusions

Cancer therapeutic response can be partially predicted on the basis of a network communicability measure that integrates gene expression and protein interaction data. This measure is influenced by central proteins in the interactome network, cancer-associated biological processes and pathways, and cancer drivers. Centered on CNA differences and by combining drugs with uncorrelated effects may help to identify on the identification of synergistic results.

## Additional files

Additional file 1: Table S1. Drugs classified as “single node” or “multiple node” perturbation. (XLSX 46 kb) Additional file 2: Table S2. Normalized CNA values for all cancer cell lines. (XLSX 55 kb) Additional file 3: Figure S1. Biological coherence of the integrated dataset. *Graph* showing the distributions of expression correlations for random gene pairs or genes that encode for physically interacting proteins. The empirical *p* value is shown. (PDF 418 kb) Additional file 4: Figure S2. Corroboration of CNA-IC_50_ correlations using a high-quality interactome dataset. The PCC distribution difference between drugs that target CTKs or STKs is maintained when the network is based on a high-quality dataset derived from the combination of reliable literature-curated binary interactions and well-verified high-throughput yeast two-hybrid interactions. (PDF 162 kb) Additional file 5: Figure S3. The CNA-IC_50_ correlation differences are not observed when a random, null network model that preserves degree distribution and connectedness is analyzed. (PDF 148 kb) Additional file 6: Table S3. Drugs with their IC_50_ negatively correlated with CNA values. (XLSX 46 kb) Additional file 7: Figure S4. Evaluation of CNA-IC_50_ correlations by known cancer driver mutations. Distributions of PCCs for cancer cell lines with mutated or wild-type proto-oncogenes or tumor suppressors as depicted in each figure. The rank position of negatively correlated drugs (*p* < 0.10) is shown. (EPS 975 kb) Additional file 8: Table S4. Correlations of drug target gene expression and CNA values. (XLSX 42 kb) Additional file 9: Figure S5.
*Top panel*, *heatmap* showing the DCIs obtained by assessing metformin combinations in two additional breast cancer cell lines. *Bottom panels*, *graphs* showing the results for metformin and/or AZD-8055. (EPS 495 kb)

## Abbreviations

CNA: Cancer network activity
DCI: Drug combination index
GO: Gene ontology HPRD, Human protein reference database
IC_50_: Half maximal inhibitory concentration
PCC: Pearson’s correlation coefficient
TF: Transcription factor
