# Supplementary figures and images for: Cancer network activity associated with therapeutic response and synergism
Source: Genome Med. 2016 Aug 24;8(1):88. doi: 10.1186/s13073-016-0340-x (PMC4995628; doi:10.1186/s13073-016-0340-x)

Figure S1

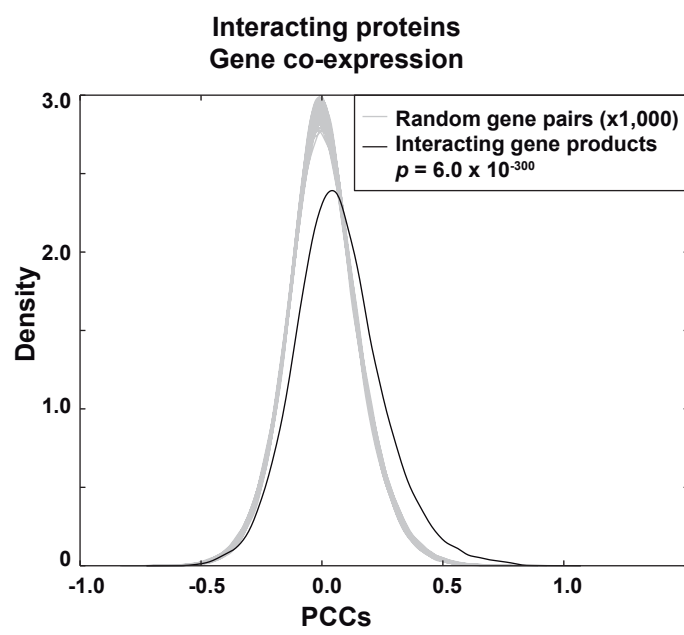

Supplement: Additional file 3: Figure S1. — Biological coherence of the integrated dataset. Graph showing the distributions of expression correlations for random gene pairs or genes that encode for physically interacting proteins. The empirical p value is shown. (PDF 418 kb) [file 13073_2016_340_MOESM3_ESM.pdf]

Figure S2

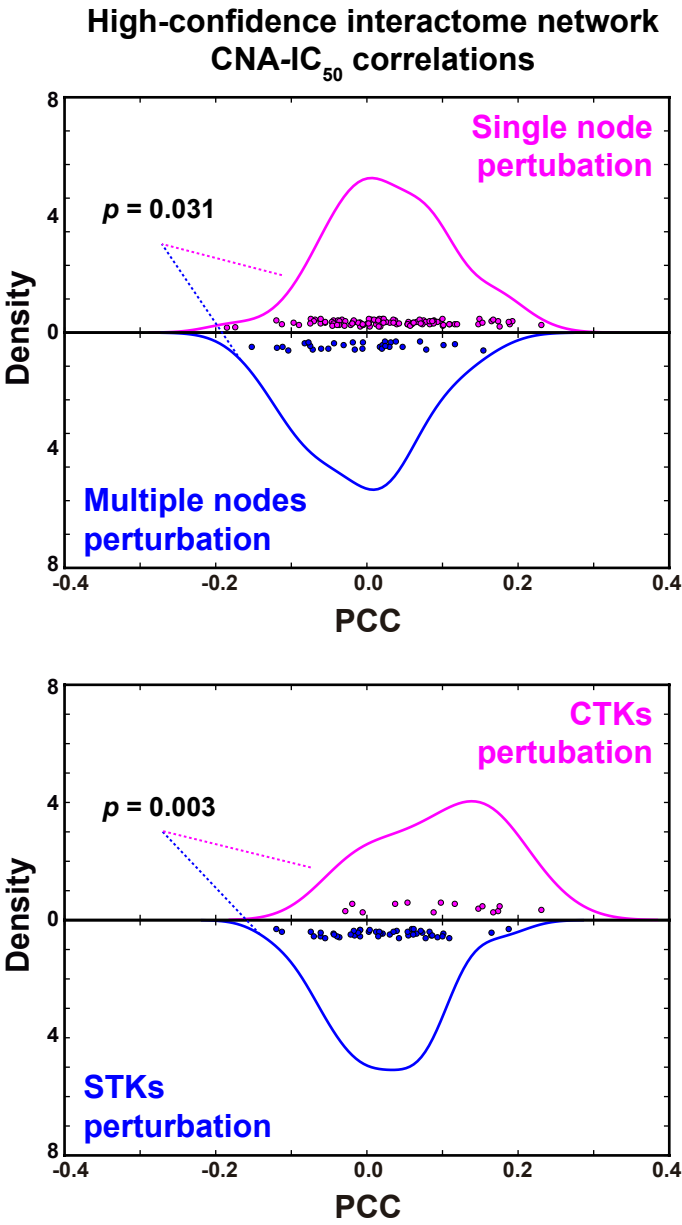

Supplement: Additional file 4: Figure S2. — Corroboration of CNA-IC50 correlations using a high-quality interactome dataset. The PCC distribution difference between drugs that target CTKs or STKs is maintained when the network is based on a high-quality dataset derived from the combination of reliable literature-curated binary interactions and well-verified high-throughput yeast two-hybrid interactions. (PDF 162 kb) [file 13073_2016_340_MOESM4_ESM.pdf]

Figure S3

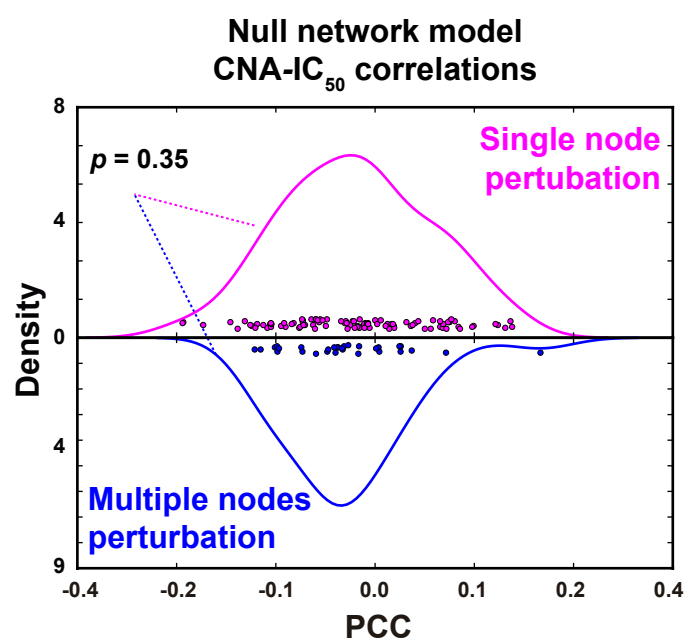

Supplement: Additional file 5: Figure S3. — The CNA-IC50 correlation differences are not observed when a random, null network model that preserves degree distribution and connectedness is analyzed. (PDF 148 kb) [file 13073_2016_340_MOESM5_ESM.pdf]
